# Statistical genetics concepts in biomass-based materials engineering

**DOI:** 10.3389/fbioe.2022.1022948

**Published:** 2022-10-04

**Authors:** Jordan Pennells, Darren J. Martin

**Affiliations:** School of Chemical Engineering, The University of Queensland, St Lucia, QLD, Australia

**Keywords:** cellulose nanofibres, nanopaper, statistical modelling, statistical genetics, heritability, hierarchical clustering, selection gradient

## Abstract

With the rise of biomass-based materials such as nanocellulose, there is a growing need to develop statistical methods capable of leveraging inter-dependent experimental data to improve material design, product development, and process optimisation. Statistical approaches are essential given the multifaceted nature of variability in lignocellulosic biomass, which includes a range of different biomass feedstock types, a combinative arrangement of different biomass processing routes, and an array of different product formats depending on the focal application. To account for this large degree of variability and to extract meaningful patterns from research studies, there is a requirement to generate larger datasets of biomass-derived material properties through well-designed experimental systems that enable statistical analysis. To drive this trend, this article proposes the cross-disciplinary utilisation of statistical modelling approaches commonly applied within the field of statistical genetics to evaluate data generated in the field of biomass-based material research and development. The concepts of variance partitioning, heritability, hierarchical clustering, and selection gradients have been explained in their native context of statistical genetics and subsequently applied across the disciplinary boundary to evaluate relationships within a model experimental study involving the production of sorghum-derived cellulose nanofibres and their subsequent fabrication into nanopaper material. Variance partitioning and heritability calculates the relative influence of biomass vs. processing factors on material performance, while hierarchical clustering highlights the obscured similarity between experimental samples or characterisation metrics, and selection gradients elucidate the relationships between characterisation metrics and material quality. Ultimately, these statistical modelling approaches provide more depth to the investigation of biomass-processing-structure-property-performance relationships through outlining a framework for product characterisation, quality evaluation, and data visualisation, not only applicable to nanocellulose production but for all biomass-based materials and products.

## 1 Introduction

Biomass-derived nanocellulose is a highly versatile material currently under investigation across a wide range of burgeoning material applications, including paper and packaging, adsorbents, cosmetics, catalysts, construction, biomedicine, and electronic devices (Pennells et al., 2020). The bio-based origin of lignocellulosic biomass significantly contributes to the sustainability of nanocellulose materials. However, lignocellulose variability is a significant challenge for the development of bio-based nanocellulose products (Zhu et al., 2016). The multifaceted nature of lignocellulose variability involves: 1) The inherent biological variability of lignocellulose from different genetic sources (plant species) and environmental growth conditions; 2) Biological variability across different tissue types within the same plant; 3) The hierarchical complexity of both the feedstock material and final product structure; and 4) Material variability attributed to differences in biomass handling, storage, processing, and product fabrication (Ciesielski et al., 2020). This variability is reflected in the versatility of nanocellulose materials, which are able to be produced from a wide variety of biomass sources (i.e., from aquatic and terrestrial biomass or through bacterial biosynthesis), generated through a range of different processing routes (i.e., enzymatic, thermal, chemical, and mechanical treatments), or fabricated into a range of different product formats (i.e., aqueous suspension or paste, dried into a networked film, spray dried into solid particles, compounded in a polymer matrix, freeze dried into an aerogel structure, or carbonized at high temperatures). Hence, this complex nature of variability must be considered when investigating biomass-derived nanocellulose materials.

Experimental system design and statistical modelling approaches that account for these sources of variability are a significantly underdeveloped aspect of the nanocellulose research field (Ramakrishna et al., 2019). Statistical approaches are most commonly utilised in nanocellulose research for process optimisation (Rojsitthisak et al., 2017; Rodrigues et al., 2019; Baruah et al., 2020; Hisham et al., 2020; Nardi et al., 2020; Mairpady et al., 2021; Lim et al., 2022; Prabu et al., 2022; Soleimanzadeh et al., 2022; Zubair et al., 2022), property evaluation (Chinga-Carrasco, 2013; Lin et al., 2014; Desmaisons et al., 2017; Kriechbaum et al., 2018; Schenker et al., 2018; Motohashi and Hanasaki, 2019; Espinosa et al., 2020; de Carvalho Benini et al., 2021; Sankhla et al., 2021), predictive modelling (Hayes, 2012; García-Gonzalo et al., 2016; Lengowski et al., 2016; Ramachandran et al., 2017; Malucelli et al., 2018; Gao et al., 2019; Ferdous et al., 2021; Demir et al., 2022), and fibre morphology estimation (Postek et al., 2011; Legland and Beaugrand, 2013; Aguado et al., 2016; Arcari et al., 2019; Ang et al., 2020; Campano et al., 2020; Uetani et al., 2021). However, statistical approaches also have the potential to be employed in alternative and creative ways. A well-structured experimental design and sample characterisation methodology can help to answer questions relating to both which biomass source and/or processing conditions generate the highest performing material, as well as the mechanisms by which high performance is achieved. This article offers a statistical approach to deal with the challenge of lignocellulose variability, through co-opting a handful of concepts from the field of statistical genetics. These statistical methodologies—including variance partitioning, heritability, hierarchical clustering, and selection gradients—have conventionally been utilised to decipher phenotypic and/or genetic variance between organisms within a species population. However, these concepts have been re-applied across disciplinary boundaries to demonstrate their utility in material research and development.

This work utilizes data from an experimental system outlined in an existing research study, involving the generation of cellulose nanofibres (CNFs) from sorghum agricultural byproducts and the subsequent fabrication of nanopaper material (Pennells et al., 2021). The factors within the experimental dataset include nanofibre samples from different: 1) sorghum varieties; 2) plant sections; 3) energy levels for mechanical fibrillation; which were subsequently fabricated into multiple 4) nanopaper material duplicates; and 5) nanopaper strip replicates per duplicate for mechanical testing. A summary of this experimental system is outlined in the Supplementary Material (Supplementary Figure S1).

The key characterisation metrics used in this study included the nanofibre-water interaction properties of sedimentation behavior and water retention value, and the mechanical properties of nanopaper material, specifically nanopaper tensile index. Sedimentation behavior was based on the sediment height of a series of diluted cellulose nanofibre samples after settling over a period of 48 h. The derivative of the sediment height trendline over the dilution series is used to calculate the estimated average nanofibre aspect ratio based on the Effective Medium Theory (Varanasi et al., 2013). The water retention value metric was based on the solids content of cellulose nanofibre samples after a standardised centrifugation process at 3,000 rpm for 10 min, which indicated the capacity for the sample to entrain water within the nanofibre network as a proxy for the specific surface area (Gu et al., 2018). Subsequent fabrication of cellulose nanofibres into nanopaper material was achieved with a Rapid Köthen handsheet former (Xell, Austria) according to ISO 5269–2:2004 (International Organization for Standardization, 2004). The mechanical properties of nanopaper material were assessed through tensile testing of multiple nanopaper strips with the Instron model 5,543 universal testing machine (Instron Pty Ltd., Melbourne, Australia), using a 500 N load cell, a crosshead speed of 1 mm/min, and a gauge length of 100 mm (Pennells et al., 2021).

## 2 Variance partitioning & heritability

In statistics, variance partitioning (or partitioning of the sums of squared deviations) involves estimating the spread of observations from the mean value for different sources of variability (or “componentsˮ) within the data set. Interestingly, the development of this field of statistics was largely driven by its application in quantitative genetics. In quantitative genetics, this concept is used to partition the variance in a phenotypic trait based on the effect of genetic and environmental components, as well as the interaction between these components. In other words, variance partitioning in this field estimates the influence of genetic and environmental factors on the phenotypic outcome of interest, a concept that is also known as heritability. In common parlance, this is the technical aspect of the “nature vs. nurture” debate. Based on the variance partitioning results, heritability can be calculated as the proportion of phenotype variance attributed to genetic factors out of the total phenotype variance for a specific trait (i.e., height).

In the context of the current biomass-based material study, the genetic component is attributed to the type of biomass feedstock utilised for CNF preparation. On the other hand, the environmental component is not attributed to the growth conditions of the biomass, as would typically be the case in heritability studies. In this case, the variance due to the growth conditions is assumed to be negligible, as the biomass was grown in the same geographical location over the same period of time. Instead, the environmental component is attributed to the laboratory processing conditions, which act as a pseudo-environment encompassing everything from biomass grinding, pulping, nanofibrillation, and nanopaper fabrication. Therefore, heritability in the context of biomass-based material engineering is a measure of the relative influence of biomass factors (i.e., sorghum variety and plant section) compared to the influence of processing conditions on the phenotypic trait value. The phenotypic trait can be selected as any property measured for the CNF or nanopaper material samples, such as a metric of nanofibre morphology, or the nanopaper tensile strength or toughness. The methodology required to partition the variance of biomass and processing components within the experimental system initially involves application of a statistical model to fit the data. In this case, a linear mixed-effects model was developed to fit the data, with the sorghum variety, plant section, and processing energy level comprising the fixed effects and the nanopaper duplicate and nanopaper strip replicates comprising the random effects. The experimental factors included within this Biomass-to-Nanopaper model are outlined in Table 1.

**TABLE 1:** Experimental factors within the Biomass-to-Nanopaper model.

Variable	Variable type	Description	Levels
$G_{i}$	Fixed, nominal	Sorghum variety	Sugargraze, Yemen, GreenleafBMR, Graingrass
${G:S}_{ij}$	Fixed, nominal	Plant section; nested within sorghum variety	Leaf, Sheath, Stem
$E_{k}$	Fixed, ordinal	Mechanical energy level (homogenisation)	Low, Medium, High
$n_{l}$	Random, nominal	Nanopaper duplicate	1–2
${n:z}_{lm}$	Random, nominal	Nanopaper strip replicate; nested within nanopaper duplicate	1–8

The response variable of the model was initially selected as the nanopaper tensile index (TI), as this metric generally indicates the quality of CNF samples for nanocomposite reinforcement applications. Therefore, the mixed-effects model is required to determine which experimental factors significantly influence the value of nanopaper tensile index value (i.e., through analysis of variance) and allow the statistical ranking of samples based on this metric (i.e., through pairwise comparison testing). However, determination of the variance attributed to the biomass factors in relation to the processing factors required the conversion of the mixed effects model into a purely random effects model. The structure of the random effects model that was implemented within the R software workspace is outlined in Eq. 1, using the lme4 package (Bates et al., 2015).

lmer(TI∼(1|variety)+(1|variety:section)+(1|energy)+(1|nanopaper/strip)) (1)

Subsequently, an analysis of variance (ANOVA) was performed on the random effects model, with the outcome yielding component variance values. The heritability scores for each component were calculated as a proportion of the overall variance, as outlined in Eq. 2, where $V_{G}$ is the variance attributed to genetic factors and $V_{E}$ is the variance attributed to laboratory “environmentˮ factors.

$$h^{2}=\frac{V_{G}}{V_{G}+V_{E}}$$ (2)

The break down of results for the partitioning of variance analysis are presented in Table 2. This statistical genetics-inspired analysis highlights that the “heritabilityˮ of the variety factor is a low as 2.2%, indicating that the type of sorghum variety had minimal relative impact on the nanopaper tensile index in comparison to the other experimental factors. On the other hand, the mechanical treatment energy level (HPH) had a significantly greater influence on the nanopaper tensile index (35.3%), highlighting the predominant effect that processing has on nanofibre and nanopaper mechanical properties. Meanwhile, the residual variance (43.8%) outweighed all other factors, emphasizing the significant degree of unaccounted for variance in the outcome of nanopaper mechanical strength.

**TABLE 2:** Partitioning of factor variance for the Biomass-to-Nanopaper random effects model for the response variable of nanopaper tensile index.

Groups	Name	Variance	Std. Dev	Factor “heritabilityˮ (%)
strip:nanopaper	(intercept)	0.01	0.11	0.004
Section:Variety	(intercept)	57.40	7.58	18.3
Variety	(intercept)	6.74	2.60	2.2
HPH	(intercept)	110.59	10.52	35.3
Nanopaper	(intercept)	1.28	1.13	0.4
Residual		137.34	11.72	43.8
SUM		313.4		100

In a similar manner, the response variable for the random effects model, which in the case above was the nanopaper tensile index, can be changed to any other CNF or nanopaper characterisation metric within the experimental system. To demonstrate an example of this, the response variable was changed to each one of the fibre morphology metrics assessed on CNF suspension samples using an automated fibre analysis device (MorFi, Techpap, France). Refer to the Supplementary Material for a full description of each of the fibre morphology parameters (Supplementary Table S1). In this case, the variance score of components (variety, section, energy level) were calculated for each of the fibre morphology parameters, as shown in the Supplementary Material (Supplementary Table S2). Utilizing these component variance scores, the ‘heritability’ of each morphology parameter was calculated as the proportion of the genetic variance scores of Section:Variety and Variety over the total parameter variance, as demonstrated in Eq. 3.

$$h^{2}=\frac{Var\left(Variety\right)+Var\left(Section:Variety\right)\;}{Var\left(Total\right)}$$ (3)

**FIGURE 1:**
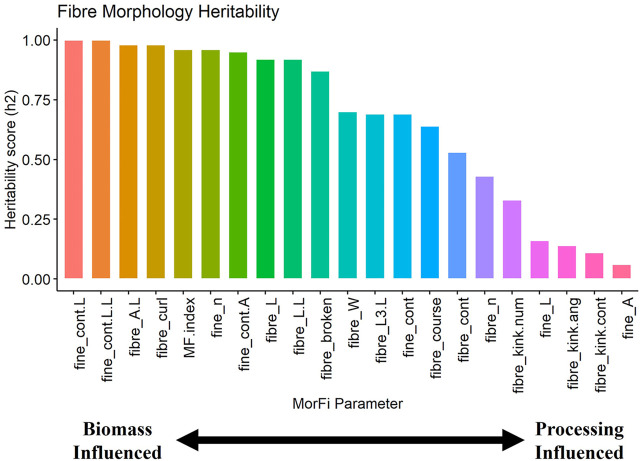
Ranking of heritability scores for each fibre morphology parameter.

In this sense, a higher heritability value indicates a fibre morphology parameter that is more influenced by biomass factors related to different sorghum varieties or plant sections, while a lower heritability value indicates a fibre morphology parameter that is more influenced by the processing factor related to the mechanical treatment energy level. Figure 1 presents the ranked heritability score of each fibre morphology parameter. Overall, this methodology of experimental design and statistical modelling is effective at untangling intertwined relationships between different factors within the experimental system and can be applied across different bio-based material research studies given a well-structured experimental design.

## 3 Phylogenetic clustering

In phylogenetics, hierarchical clustering is a statistical method used to evaluate the genetic similarity between a set of different genes, species, or other taxa, typically visualized in the form of a branching tree diagram. The data used to generate the relationship between samples in the form of phylogenetic trees (i.e., dendrograms) is typically measured phenotypic data, or more recently on the nucleotide sequence of genes. However, this statistical method of measuring similarity between samples can be applied to a wide variety of different data set types, including for CNF morphology generated in the experimental system visually outlined in the Supplementary Material (Supplementary Figure S1). In this case, hierarchical clustering can be used to evaluate the similarity between each CNF sample within the experimental series based on the amalgamation of all fibre morphology parameters (Supplementary Figure S2a), or alternatively to evaluate the similarity of different fibre morphology parameters based on all CNF samples (Supplementary Figure S2b).

Interestingly, the groupings generated for both the biomass sample and fibre morphology parameter clustering were highly intuitive and presented informative groups. For biomass sample clustering, this approach readily distinguishes the variability in fibre morphology for the leaf section of sorghum biomass (Supplementary Figure S2a, Cluster 2 + 4). In addition, the characterisation metric clustering convincingly delineates the different types of fibre morphology characteristics, such as the fine content parameters (Supplementary Figure S2b, Cluster 1), fibre kink parameters (Supplementary Figure S2b, Cluster 2), and fibre length parameters (Supplementary Figure S2b, Cluster 3). Therefore, this methodology has the potential to provide deeper insights into the relationships between different experimental samples, in addition to drawing links between the relationship of different characterisation methods or sample properties.

## 4 Selection gradients & predictive evolution

In statistical genetics, the Breeder’s equation is a statistical tool utilised to predict natural microevolution or the inter-generational effects of selective breeding. The multivariate form of the equation can include a collection of phenotypic traits of a species population, mapping the relationship between traits to the fitness score for the population from generation to generation. This information is sufficient to predict the evolutionary shift in the average value of each measured phenotype over one generation of reproduction or breeding. The Breeder’s equation, also known as the Lande Equation, is shown in Eq. 4 below.

$$\Delta \bar{z}=G\beta$$ (4)

In expanded form, the multivariate Breeder’s equation can also be written as in Eq. 5, where $\Delta {\bar{z}}_{n}$ is the change in the value of phenotypic trait n from generation 1 to 2, G is the variance-covariance matrix for all phenotypic traits included in the model, and β is the selection gradient matrix describing the relationship between each phenotypic trait and fitness.

$$\left(\begin{array}{c} \Delta {\bar{z}}_{1}\\ \Delta {\bar{z}}_{2}\\ \ldots \\ \Delta {\bar{z}}_{n} \end{array}\right)=\left(\begin{array}{cccc} V_{1} & Cov_{1,2} & \ldots & Cov_{1,n}\\ Cov_{2,1} & V_{2} & \ldots & Cov_{2,n}\\ \ldots & \ldots & \ldots & \ldots \\ Cov_{n,1} & Cov_{n,2} & \ldots & V_{n} \end{array}\right)\;\left(\begin{array}{c} \beta_{1}\\ \beta_{2}\\ \ldots \\ \beta_{n} \end{array}\right)$$ (5)

While the production of CNF from biomass feedstock is not an evolving system, which prevents the complete application of the Breeder’s equation in this context, some aspects of this methodology can be applied for biomass-derived material research. For example, while each row within Eq. 5 would typically represent a different phenotypic trait of the species population, in the case of material processing each row can represent a different characterisation metric for the CNF sample population. The variance of each metric ( $V_{n}$ ) represents the standardised spread in that metric value across the entire sample population, while the covariance ( $Cov_{n,m}$ ) represents the relationship between metric n and metric m .

This methodology becomes interesting when considering the role of the selection gradient $\beta_{n}$ , which in the context of quantitative genetics represents the relationship between the phenotypic trait and the evolutionary fitness of the organism. The selection gradient value is simply the slope of the linear regression between the phenotypic trait values for all individuals within the population and their fitness score, indicating the positive or negative relationship to evolutionary fitness with an increased trait value. However, in material processing, fitness can be considered as the overall quality of the material sample. Along this thread, our recent research article has outlined a novel method to distil multiple characterisation metrics into a single quality score value, in a flexible and tunable manner that is valid for any material application (Pennells et al., 2022). The quality score acts as a proxy for evolutionary fitness in this context. Therefore, the “fitnessˮ (or quality) of material samples varies depending on the selected definition of quality, based on the focal application, whereas the definition of fitness in the evolutionary sense is more constrained to the reproductive success of the individual organism.

**FIGURE 2:**
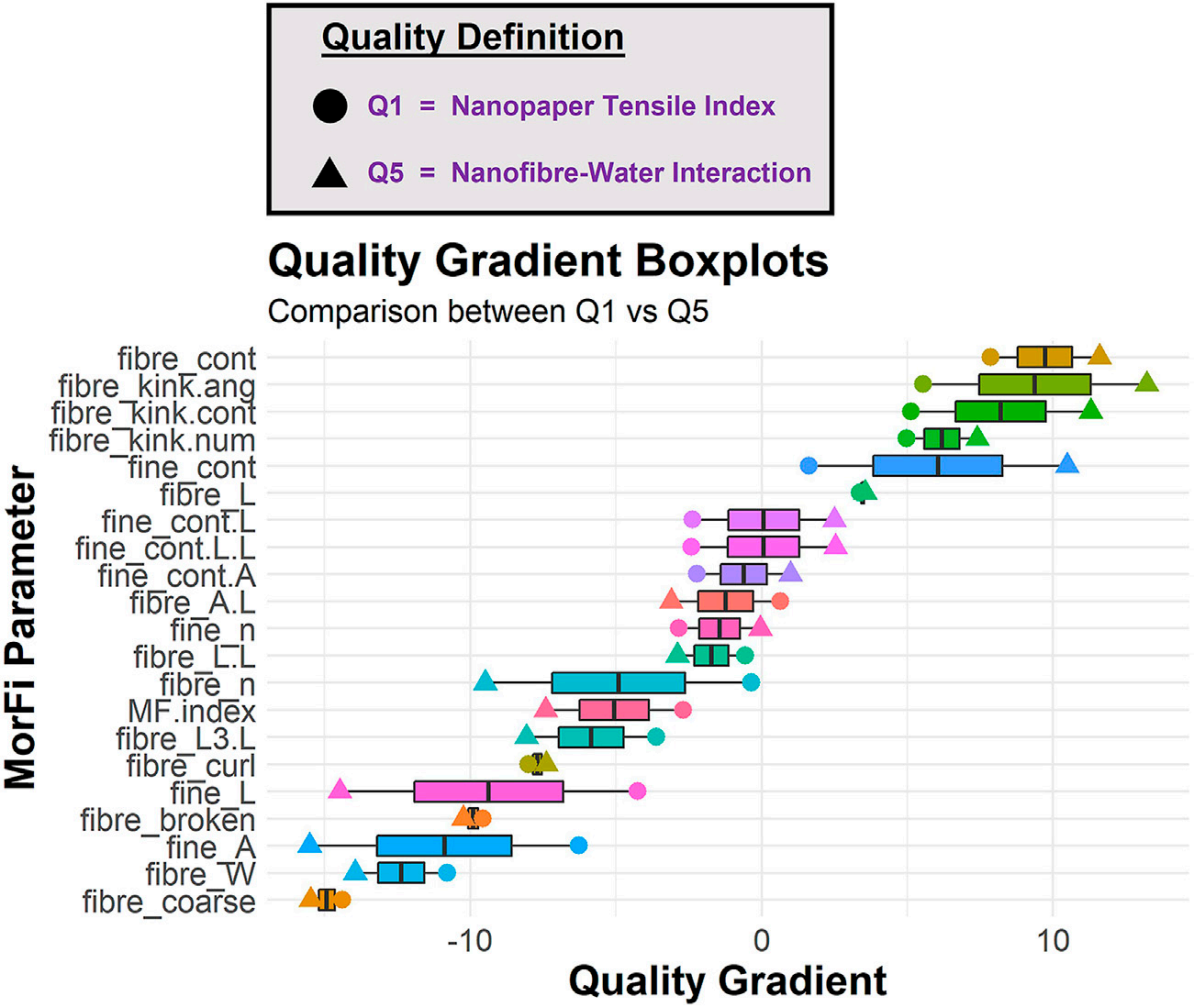
Comparison of biomass fibre morphology selection gradients across the Q1 (nanopaper tensile index) and Q5 (CNF sedimentation aspect ratio and WRV) quality definitions.

Utilizing this tunable property of quality in the context of material engineering, multiple selection gradient values can be generated for each characterisation metric as specified by different quality definitions. The utility of this approach for CNF quality evaluation is demonstrated in Figure 2, where the selection gradient values for each fibre morphology metric are compared across two different quality definitions. Quality 1 (Q1) represents the sole definition of the nanopaper tensile index as the quality indicator, while Quality 5 (Q5) represents the nanofibre-water interaction properties of CNF sedimentation ratio and water retention value (Pennells et al., 2022). Each “box plotˮ within Figure 2 consists of two data points, which represent the Q1 and Q5 selection gradient values for each fibre morphology parameter. Refer to the Supplementary Material for a full description of each of the fibre morphology parameters.

This diagram presents three key pieces of information: 1) The ascending order of the mean value between the selection gradient values for the two quality definitions (represented as the middle line of each box plot) signifies how the fibre morphology parameter influences quality on average across the two contexts. For example, fibre coarseness (fibre_coarse) has a strong negative selection gradient (i.e., a negative relationship with sample quality) for both the Q1 and Q5 definitions, indicating that a high fibre coarseness generates a low quality material in both the fibre-fibre and fibre-water interaction strength situations. On the other hand, a high fibre content (fibre_cont) generates a high quality material for both of these situations; 2) The fibre morphology parameters that are situated in the middle of the diagram (i.e., fibre_L.L, fine_n, fibre_A.L, fine_cont.A, fine_cont.L.L, fine_cont.L) exhibit a minimal impact on product quality for both situations; and 3) The spread of the box plot indicates how much the selection gradient value changes across the two quality definitions. For example, fibre length (fibre_L) has a very similar influence on product quality for both fibre-fibre and fibre-water interaction applications, whereas the fine content (fine_cont) has a divergent influence on product quality depending on the application, such that a high fine content sample generates a high quality material for the fibre-water interaction application but significantly less so for the nanopaper strength application.

Overall, this quantitative genetics-inspired methodology provides a new approach to visualizing the effect of characterisation metrics on nanocellulose material quality across multiple focal applications. Utilizing the selection gradient values in concert with the metric variance and covariance values, as outlined in Eq. 5, $\Delta {\bar{z}}_{n}$ values can be calculated for all characterisation metrics. In this context, these values would represent the true relationship that each characterisation metric has with material quality, taking into account the degree of variation exhibited throughout the population and the correlation with other metrics that may negatively impact quality.

## 5 Conclusion

This article outlines a novel approach to experimental data analysis and visualisation for biomass-based material processing, utilizing a combination of statistical methods commonly applied in quantitative genetics. Variance partitioning and heritability estimation (of the experimental system) presents the opportunity to uncover the relative influence of biomass or processing factors on the final material performance, given a well-structured experimental design. Furthermore, the relative influence of biomass or processing factors can be estimated for each CNF characterisation metric, which in this case was demonstrated for the fibre morphology parameters. The relationships between characterisation metrics can also be assessed through a phylogenetics approach, which estimated the relative similarity of samples or characterisation metrics within the data set and exhibited intuitive clustering of the fibre morphology parameters. Lastly, a statistical approach commonly employed for predictive evolution and selective breeding studies was utilised to generate selection gradient values outlining the relationship between each characterisation metric and the quality score of samples within the experimental population, which serves as a proxy for evolutionary fitness. This approach enables visualisation of the characterisation metric influence on sample quality, when quality can be defined in different ways depending on the product type or focal application. The importance of these statistical approaches resides in their versatility across different material types—these methods are not limited to nanocellulose materials but can be applied across different studies that involve the processing of biomass feedstock for the production of various bio-based materials or products. Overall, integration of statistical genetics concepts into the field of biomass-based material engineering has the potential to increase the depth of investigation into the factors that influence sample quality, the relationships between characterisation metrics, and creates the opportunity for incorporation of genetic data of biomass feedstock in sustainable material investigation, all of which are essential for the industrial scale-up of highly variable and difficult to quantify, and qualify, nanocellulose materials.

## Data Availability

The datasets presented in this study can be found in an online repository. The name of the repository can be found below: https://github.com/jordanpennells/PhD-sorghum.git.
