# Supplementary material for: Statistical genetics concepts in biomass-based materials engineering
Source: Front Bioeng Biotechnol. 2022 Oct 4;10:1022948. doi: 10.3389/fbioe.2022.1022948 (PMC9577247; doi:10.3389/fbioe.2022.1022948)
Supplement: Supplementary file 1 [file DataSheet1.docx]

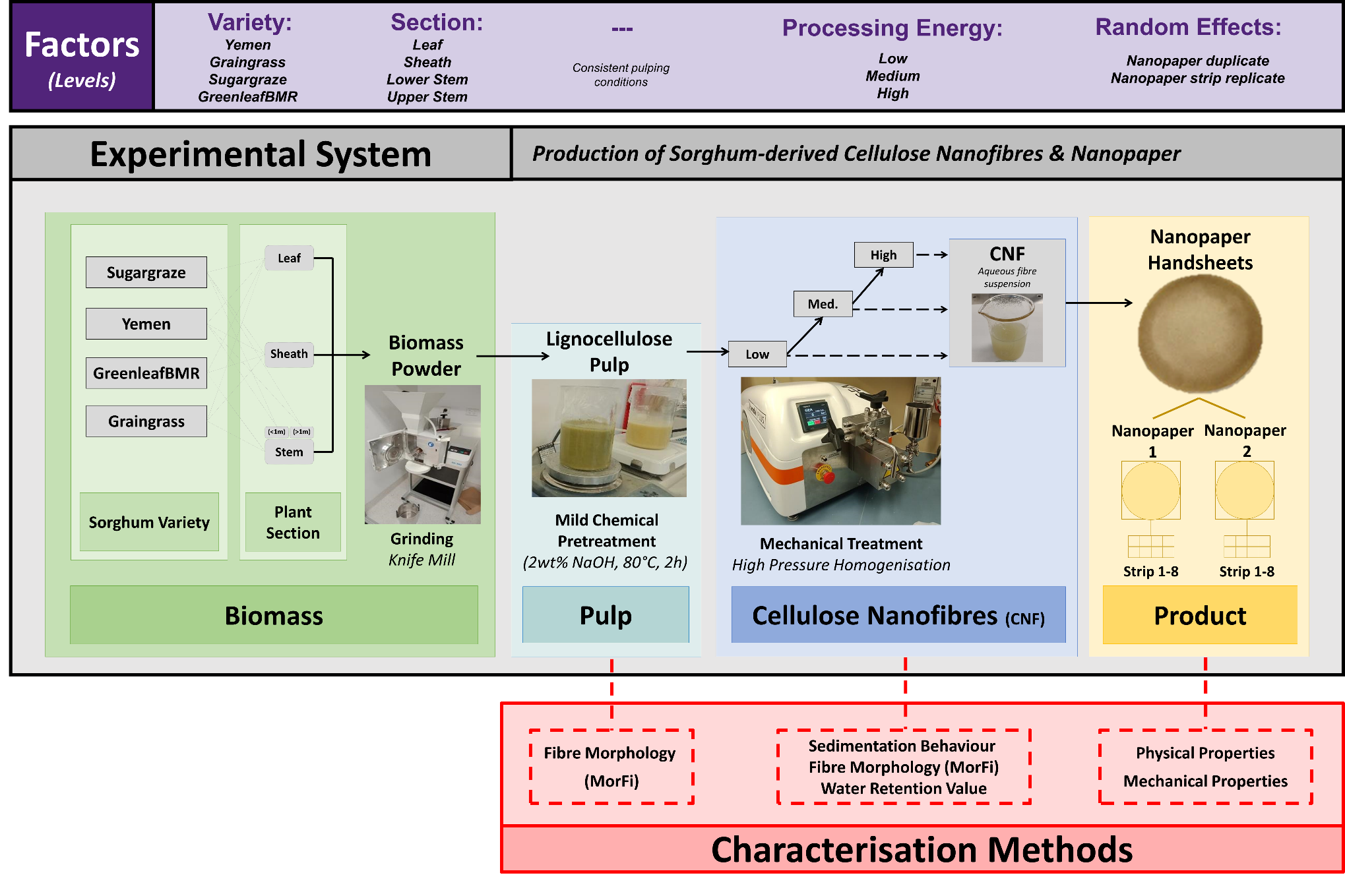
Supplementary Material

Supplementary Figure S1 Graphical representation of the experimental system for Biomass-to-Nanopaper modelling.

Supplementary Table S1 MorFi output parameters and parameter descriptions

| Code | Parameter | Unit | Description |
| --- | --- | --- | --- |
| fibre_n | Number of analysed fibres | - | The total number of fibres analysed throughout the test |
| fibre_cont | Fibre content | millions/g of pulp | The number of fibres (millions) per gram of sample analysed |
| fibre_L | Mean arithmetic length | µm | Arithmetic mean length of all fibres analysed |
| fibre_L.L | Mean length-weighted length | µm | Arithmetic mean length of all fibres analysed, weighted by fibre length |
| fibre_L3.L | Mean cubic-length-weighted length | µm | Arithmetic mean length of all fibres analysed, weighted by the cube of the fibre length |
| fibre_A.L | Mean area-weighted length | µm | Arithmetic mean length of all fibres analysed, weighted by fibre area |
| fibre_W | Mean fibre width | µm | Arithmetic mean width of all fibres analysed |
| fibre_coarse | Mean fibre coarseness. mg/m | mg/m | Fiber mass per unit length |
| fibre_kink.num | Mean kink number | - | The number of fibres with small regions of very high curvature (i.e. sharp bends) along the fiber. |
| fibre_kink.ang | Mean kink angle | ° | The mean kink angle for fibres that are kinked |
| fibre_kink.cont | Kinked fibre content | % | The number percentage of fibres with a kinked structure |
| fibre_curl | Mean fibre curl index | % | Degree of nonstraightness of a fiber - or the ratio of total particle length over the projected length |
| MF.index | MacroFibrillation index | % | Percentage of microfibrils by area |
| fibre_broken | Broken fibre content | % | Number percentage of fibres with broken ends |
| fine_n | Number of analysed fines | - | The total number of fibres analysed throughout the test |
| fine_cont | Fine content | millions/g of pulp | The number of fines (millions) per gram of sample analysed |
| fine_cont.A | Fine content | % (in area) | % Area of Fine Elements over all other elements |
| fine_cont.L | Fine content | % (in length) | % Length of Fine Elements over all other elements |
| fine_cont.L.L | Fine content | % (in length-weighted length) | % Length weighted length of Fine Elements over all other elements |
| fine_A | Mean fine area | µm² | Arithmetic mean fine area |
| fine_L | Mean fine length | µm | Arithmetic mean fine length |

Supplementary Table S2 Variance partitioning values for each fibre morphology parameter

| **Parameter** | **Variance Score** | | | | |
| --- | --- | --- | --- | --- | --- |
|  | **Section: Variety** | **Variety** | **Energy Level** | **Total** | **h^2^** |
| *fine_cont.L* | 0.6 | 0 | 0 | 0.6 | **1.00** |
| *fine_cont.L.L* | 0.71 | 0 | 0 | 0.71 | **1.00** |
| *fibre_curl* | 0.44 | 0.42 | 0.02 | 0.88 | **0.98** |
| *fibre_A.L* | 0.81 | 0.02 | 0.02 | 0.85 | **0.98** |
| *MF.index* | 0.65 | 0 | 0.03 | 0.68 | **0.96** |
| *fine_n* | 0.59 | 0 | 0.03 | 0.62 | **0.95** |
| *fine_cont.A* | 0.74 | 0 | 0.04 | 0.78 | **0.95** |
| *fibre_L* | 0.64 | 0 | 0.05 | 0.69 | **0.93** |
| *fibre_L.L* | 0.83 | 0 | 0.08 | 0.91 | **0.91** |
| *fibre_broken* | 0.41 | 0.34 | 0.11 | 0.86 | **0.87** |
| *fibre_W* | 0.45 | 0.22 | 0.28 | 0.95 | **0.71** |
| *fibre_L3.L* | 0.66 | 0 | 0.3 | 0.96 | **0.69** |
| *fine_cont* | 0.59 | 0 | 0.27 | 0.86 | **0.69** |
| *fibre_course* | 0.23 | 0.34 | 0.32 | 0.89 | **0.64** |
| *fibre_cont* | 0.13 | 0.36 | 0.43 | 0.92 | **0.53** |
| *fibre_n* | 0.24 | 0 | 0.31 | 0.55 | **0.44** |
| *fibre_kink.num* | 0.2 | 0.03 | 0.46 | 0.69 | **0.33** |
| *fine_L* | 0.15 | 0 | 0.77 | 0.92 | **0.16** |
| *fibre_kink.ang* | 0.13 | 0 | 0.78 | 0.91 | **0.14** |
| *fibre_kink.cont* | 0.09 | 0 | 0.78 | 0.87 | **0.10** |
| *fine_A* | 0.06 | 0 | 0.83 | 0.89 | **0.07** |
| **Average** | **0.45** | **0.08** | **0.28** | **0.81** | **0.67** |

Supplementary
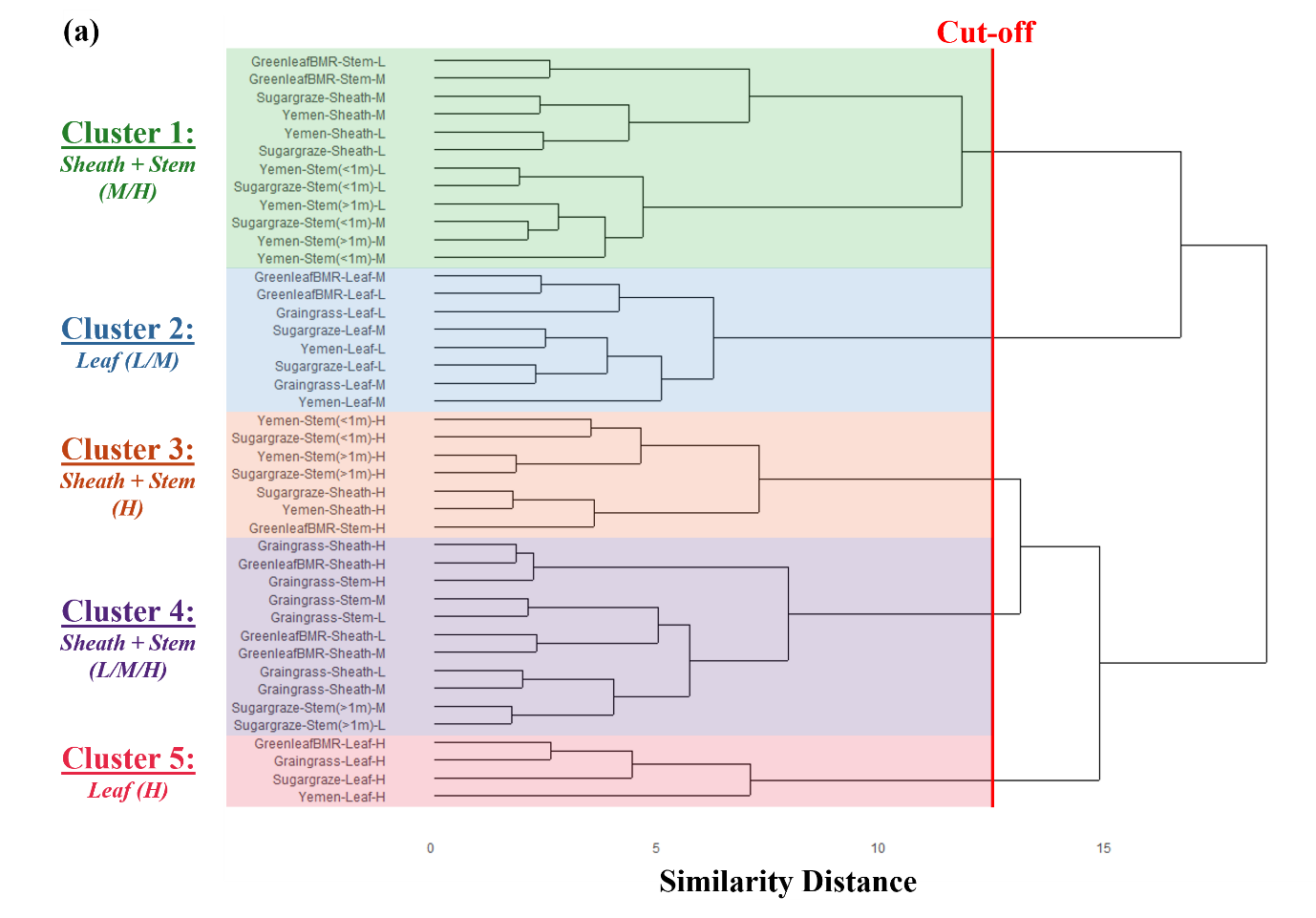

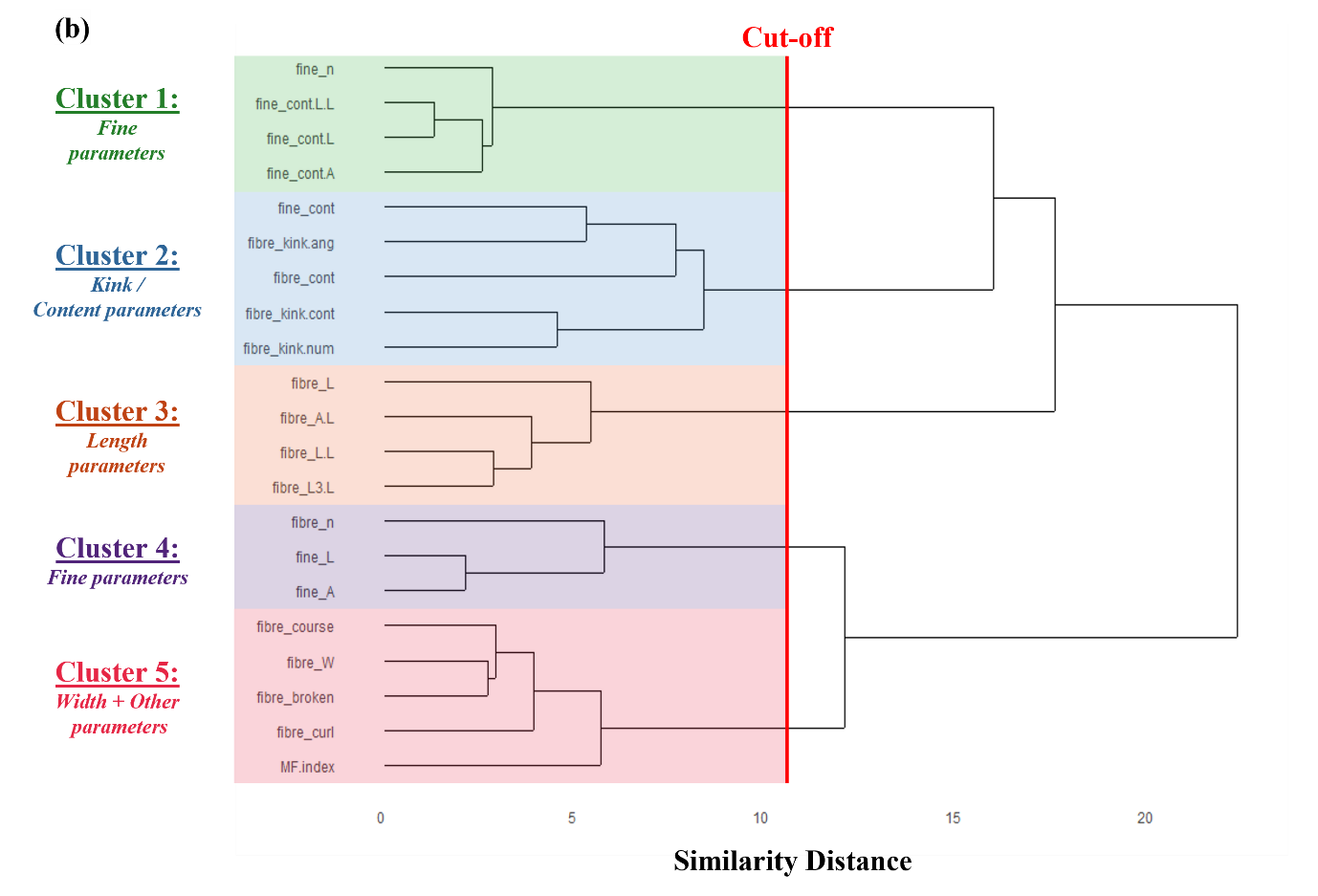
Figure S2 Hierarchical clustering dendrograms of fibre morphology data based on the: (a) Sorghum biomass samples, and (b) Fibre morphology parameters.
